# Why Antibiotic Use Data in Animals Needs to Be Collected and How This Can Be Facilitated

**DOI:** 10.3389/fvets.2017.00213

**Published:** 2017-12-12

**Authors:** Jorge Pinto Ferreira

**Affiliations:** ^1^Safoso AG, Liebefeld, Bern, Switzerland

**Keywords:** AMR, AMU, incentives, public health, data collection

## Abstract

Antimicrobial resistance (AMR) is currently recognized as one of the most significant threats to public health worldwide. It is a phenomenon that highlights the interconnectivity between human and animal health since any use of antibiotics in humans can eventually lead to resistance in the microbial populations colonizing animals and *vice versa*. In recent years, our understanding of the relationship between the use of antibiotics and the consequent development of resistance in microbial populations to these (or similar) antibiotics has increased. Having accurate data, ideally in a digital format, on the use of antibiotics are therefore of paramount importance. Current obstacles to having such data include, among others, the lack of consensual and harmonized technical methods and units that represent antimicrobial use (AMU), the insufficient incentives to motivate primary producers to report their use of antibiotics, and the inexistence of user-friendly technologies for the collection of such data, despite the generalized use of Internet and electronic devices. Further development and adoption of the units proposed by the European Surveillance of Veterinary Antimicrobial Consumption will contribute to the long-desired harmonization. Rewarding the animal producers (via tax incentives, for example) that use less antibiotics and the development of an app, to which producers could orally report the used antibiotics are among the solutions that could help to overcome the current challenges. I here also argue that having mandatory electronic veterinary prescriptions and awareness campaings, funded via public–private partnerships, should also be considered as methods that could help for the control of societal problems like AMR.

## Introduction

The discovery, availability, and use of antibiotics (antimicrobials in a broader sense) have had a major positive impact on the development and progress of human medicine in the past decades (1). Similarly, antibiotics have also significantly decreased the morbidity and mortality of animals, therefore revolutionizing animal production (2, 3). However, this “golden age” seems to be coming to an end (4), with an increasing number of reports highlighting alarming levels of antimicrobial resistance (AMR), including resistance to last resort options (5), and very few new classes of antibiotic being commercialized by the pharmaceutical industry (6). Thus, the range of antimicrobials currently available for use, with little risk of resistance affecting treatment, is dwindling.

Antimicrobial resistance is a natural phenomenon (7). As most antibiotics are derived from natural sources, microorganisms have been exposed to them throughout evolution; the development of resistance is therefore a natural survival strategy (8). However, the alarming levels of resistance, reported worldwide in animals (9, 10), humans (11, 12), and the environment (13–15), is generally agreed to be a consequence of the massive use of antibiotics in both humans and animals (5, 16) and is also strongly affected by environmental regulator factors (17, 18). The economic importance of AMR is also substantial (19, 20). Therefore, having accurate and easy to analyze data on antibiotic use is of critical importance, as a step to be able to identify the main specific primary drivers for the downstream development of resistance and tackle them.

## The Use of Digital Data on Antibiotics Utilization

Having (digital) data on antimicrobial use (AMU) would allow for:

- Differentiation of antibiotic use by species treated: at the moment, in the vast majority of countries, it is not yet possible to identify in which species a specific antibiotic has been used. Currently, antibiotics targeting multiple animal species are licensed to be sold with the same commercial name (21). This, in turn, limits the usefulness of sales data as it is therefore not possible to know in which species a specific antibiotic that was sold was used (22). Yet having this information is critical to be able to do source attribution, whereby the ultimate goal is to be able to know, which use, and in which species led to the development of resistance. Once this is possible, risk management options can be implemented;
- Identification of good and best practices: being able to identify quantitatively which practices (either at the individual producer level or at the national level) lead to a reduced AMU, while ideally keeping the same productivity in animal rearing (23), is critical. These good practices can then be promoted, through policy change based on a solid evidence base (24);
- Identification of a temporal association between AMU and AMR; this is particularly useful when use of a specific product/antibiotic is terminated, either on a voluntary basis (eg. farmers or clinicians) or as a result of a legal ban (25); it is in fact crucial to validate the implementation of such legislative measures, that can also target the use of, for example, heavy metals (like silver, copper, and zinc), that due to co-selection and cross-resistance mechanisms, eventually can also lead to resistance to antibiotics (26). Moreover, there is now an intense debate around how long an antibiotic course should ideally be (10, 27), and these data would contribute to clarifying this critical aspect.
- Evaluation of specific policies that, for example, target the reduction of AMU: an example of such a policy would be the “yellow card policy” first implemented in Denmark. Within the scope of this policy, farmers are alerted when their use of antibiotics is above a set threshold based on what would be expected (28). Evaluation of these policies would then allow for their implementation to be adjusted accordingly (9, 25).

In the past two decades, we have seen the advent and generalized use of computers, cell phones, and a range of other electronic devices, together with the booming of the Internet. This technology provides a route to unprecedent data access. Data related to AMU in livestock production, for example, could be made open access and thus readily available online for all interested stakeholders (such as policy makers, veterinary services, etc.). However, this is, unfortunately, not yet the case.

Considering the usefulness of AMU data, the availability of technology, and the higher educational levels of the younger livestock producers, it seems that at least some of the major requirements for increased availability of digital AMU are available in more economically developed countries. However, at the moment, very few countries actually have automated digital data collection for AMU (29). Therefore, most current analyses on AMU are based on sales data which have significant limitations as described previously, to which it can be added the fact that the sale of an antibiotic does not provide any information about its actual use (i.e. how, when, where, by whom). It is therefore important to analyze the reasons that can potentially explain this gap between what would be expected (having digital AMU data) and the reality (most frequently, the best available data is sales data).

## Current Obstacles and Limitations

A critical initial question is: has the scientific community reached a mature and consensual decision regarding which data should be collected and how these should be recorded? Unfortunately, the answer is “not yet” (30). The harmonization and standardization of units and methods to record AMU have long been a goal and pursuit of the scientific community (31, 32). Yet at the European level, for example, the animal species for which AMU data is currently collected are quite diverse, with some countries collecting information for all species, while others only collecting it just for the major livestock species (such as pigs or cattle). Furthermore, the technical unit used to measure AMU at the European level can be the Animal Daily Dose (ADD), the Defined Daily Dose (DDD), or simply mg, while the indicators can be, among others, mg/Population Correction Unit (PCU), mg/kg, “Treatment frequency,” or “Therapy index” (29).

Although it might seem contradictory, despite the above, Europe can arguably be seen as the leading region (33), in working toward the harmonization of methods. The significant progress and milestones achieved by the European Surveillance of Antimicrobial Consumption (ESVAC) project (such as the publication of the list of Defined Daily Dose (DDDvet)) (34) should be highlighted and recognized. Hopefully, these publications can be used as a guideline or template in other regions of the globe.

Besides the absence of consensual and harmonized units and methods, other factors have also limited the availability of AMU information:

(i) the right incentives to motivate farmers to record on a digital format their AMU data have not yet been found, thus greatly limiting the amount and quality of data that can be accrued from these primary stakeholders;
(ii) at the moment, much of the attention is given to identify those producers that use more antibiotics (35), which might trigger fear for potential penalties and represent another obstacle to have AMU data;
(iii) despite the plethora of available technology, there is still no appropriate technology that allows for the recording of AMU data in an easy and fast way.

## Which Solutions Could Therefore be Implemented to Improve the Currently Non-Satisfactory Reality?

Solutions designed to collect producer AMU information (such as online platforms, apps, etc.) need to be user-friendly and tailored to the different circumstances (species, countries, languages). Additionally, it is important to include a wide range of different professionals [from IT requirement engineers to social scientists (36, 37)] right from the start to ensure that the final product meets its purpose, and has the appropriate medical and pharmaceutical framework behind it. An example of a solution could be a mobile app, that would translate verbal data from producers regarding a specific antibiotic treatment performed, into digital data on AMU. This solution would have also to accommodate the specific use of in-feed antibiotics (38–40), namely species, age group and number of animals fed.

The development of these tools needs to be implemented together with capacity building through educational programs and tailor-made training for the producers themselves to overcome any potential initial resistance or concerns they may have regarding the adoption of newtechnology.

A critical starting point should not be underestimated: if the drugs available on farm can only be purchased after an electronic veterinary prescription, this will already provide data about the “initial pool” of the drugs/antibiotics present/available at a farm (41). The next step should be the collection and recording of the information about the actual use, for example: species, age group and number of animals fed, when considering, for example, in-feed antibiotic use.

Having these (mandatory) electronic veterinary prescriptions has several prerequisites: the producer and the veterinarian must first establish a solid and trustful professional relationship, ultimately translated into an actual written contract (vs. an emergencies-based veterinary assistance). As part of this contract, veterinarians should be requested to provide (economic) feedback on the collected data, with the goal of maximizing the economic return of the farm – this will represent a major incentive for farmers to contribute to the AMU electronic data collection, giving them also an important sense of actual ownership and access of the data.

Awareness campaigns will also be another part of the solution. These campaigns should highlight the connection between AMU data and human health, by expounding the concept that providing animal AMU data not only has a direct effect and impact on animal health, but also has spill-over effects on human (16) and environmental health (13). Financing such campaigns can be challenging. But AMR is a societal problem (42), for which I argue, public-private funding partnerhsips should be developed out of the best interest of both parts; if it is true that the use of antibiotics/antimicrobials is mostly done in the private sector (particulary on animals) the development of resistance in humans, from animal origin, eventually leads to very significant economic expenses by the different public health authorities (43). And in reality, transmission of pathogens carrying AMR determinants can also happen in the human–animal direction (44, 45). The work by Höjgård et al. (46) suggests a societal net benefit, in the specific case of the prevention of the introduction of Livestock-associated Methicillin-resistant *Staphylococcus aureus* (LA-MRSA) into Sweden and subsequent prevention of human infections (46).

Regardless of the solutions (to increase digital AMU data availability) proposed, the likelihood of their successful uptake will certainly be increased if a bottom-up approach is adopted, whereby the livestock producers’ opinions are heard and taken into consideration. Listening to all of them is obviously unrealistic, but umbrella organizations can aggregate their views and express them accordingly.

I here argue that some of the solutions that can contribute to merging the existing gap, between what would be expected, and what is the reality, when it comes to the availability of AMU digital data include:

- further developments of the harmonization strategy that ESVAC is pursuing;
- having veterinary electronic prescriptions of the drugs available on a farm;
- requiring the existence of a consultancy contract between farmers and their veterinarians, as well as between feed manufacturers and veterinarians;
- creating the (financial) incentives that can enhance farmers’ motivations to keep digital data on their AMU;
- awareness campaigns highlighting the relation between AMR in humans and animals and the consequent usefulness of AMU data;
- the development of user-friendly technological options.

Data are increasingly seen as the 21st century gold and, if collected and analyzed in the proper way, they can indeed contribute to our understanding and control of a societal problem such as AMR.

## Author Contributions

Jorge Pinto Ferreira is a Doctor of Veterinary Medicine with five years of clinical experience (food animals); a Masters in Food Safety; a PhD (as Fulbright scholar) in Public Health with a graduate certificate in Public Policy (NCSU & Duke); and a recent Diplomate of the European College of Veterinary Public Health. Jorge currently works as aconsultant at SAFOSO AG, where he has been dedicating most of his professional career to AMR. He attended the AHEAD 2017 workshop, where he presented some of the capacity building work that SAFOSO did/is doing in countries like Vietnam and Ukraine. In this perspective paper, he presents his own perspective about the different aspects related with Antimicrobial Usage (AMU), particularly the reasons behind the availability (or lack of it) of harmonized electronic digital data, and some potential solutions to overcome this.

## Conflict of Interest Statement

JF, is a consultant at SAFOSO, a private company, based in Bern, Switzerland, that routinely is engaged in different research projects [including about AntiMicrobial Resistance (AMR)], funded by for example the European Union, and similar funding bodies.
